# Friends in high places: Interspecific grooming between chimpanzees and primate prey species in Budongo Forest

**DOI:** 10.1007/s10329-023-01053-0

**Published:** 2023-02-15

**Authors:** Elodie Freymann, Michael A. Huffman, Geresomu Muhumuza, Monday Mbotella Gideon, Klaus Zuberbühler, Catherine Hobaiter

**Affiliations:** 1 Primate Models for Behavioural Evolution Lab, Department of Anthropology and Museum Ethnography, Institute of Human Sciences, University of Oxford, Oxford, UK; 2 Wild Minds Lab, School of Psychology and Neuroscience, University of St Andrews, St Andrews, UK; 3 Wildlife Research Center, Inuyama Campus, Kyoto University, Inuyama, Japan; 4 Budongo Conservation Field Station, Masindi, Uganda; 5 Department of Comparative Cognition, Institute of Biology, University of Neuchâtel, Neuchâtel, Switzerland

**Keywords:** Interspecific interactions, Interspecific grooming, Polyspecific interactions, *Pan troglodytes*, Guenon

## Abstract

While cases of interspecies grooming have been reported in primates, no comprehensive cross-site review has been published about this behavior in great apes. Only a few recorded observations of interspecies grooming events between chimpanzees and other primate species have been reported in the wild, all of which have thus far been in Uganda. Here, we review all interspecies grooming events recorded for the Sonso community chimpanzees in Budongo Forest Reserve, Uganda, adding five new observations to the single, previously reported event from this community. A new case of interspecies play involving three juvenile male chimpanzees and a red-tailed monkey is also detailed. All events took place between 1993 and 2021. In all of the six interspecific grooming events from Budongo, the ‘groomer’ was a female chimpanzee between the ages of 4–6 years, and the ‘recipient’ was a member of the genus *Cercopithecu*s. In five of these events, chimpanzee groomers played with the tail of their interspecific grooming partners, and except for one case, initiated the interaction. In three cases, chimpanzee groomers smelled their fingers after touching distinct parts of the receiver’s body. While a single function of chimpanzee interspecies grooming remains difficult to determine from these results, our review outlines and assesses some hypotheses for the general function of this behavior, as well as some of the costs and benefits for both the chimpanzee groomers and their sympatric interspecific receivers. As allogrooming is a universal behavior in chimpanzees, investigating the ultimate and proximate drivers of chimpanzee interspecies grooming may reveal further functions of allogrooming in our closest living relatives, and help us to better understand how chimpanzees distinguish between affiliative and agonistic species and contexts.

**Supplementary Information:**

The online version contains supplementary material available at 10.1007/s10329-023-01053-0.

## Introduction

Anecdotal observations suggest that many chimpanzee communities engage with sympatric primate species through a variety of interactions ranging from agonistic to affiliative—with community-specific and individual variation in behavioral responses (Teleki 1973; Hobaiter et al. 2017). Aggressive agonistic interspecific interactions appear to be the best documented, including interactions driven by competition (Morris and Goodall 1977; Matsumoto-Oda 2000), predation through hunting (Nishida et al. 1979; Boesch and Boesch 1989; Stanford et al. 1994; Uehara 1997; Mitani and Watts 2001; Teelen 2008; Newton-Fisher et al. 2002; Hobaiter et al. 2017), or possibly a combination thereof (e.g., during territorial boundary patrols; Southern et al. 2021). Both types of interactions can include chasing, physical contact (Brown and Crofoot 2013), or lethal aggression (Southern et al. 2021). Less active forms of aggressive agonism are also common, including facial expressions, threatening vocalizations, or displays (Brown and Crofoot 2013). Potentially neutral interactions, including co-feeding, have also been reported (Hosaka and Ihobe 2015), in which chimpanzees were observed ignoring prey species in feeding contexts, despite the prey’s proximity and capturability. Affiliative interspecific interactions in chimpanzees have also been observed in the wild including play (Goodall 1986; Teleki 1973), and grooming (see Tsutaya et al. 2018; Bakuneeta 1996; John and Reynolds 1997).

Chimpanzees share their home ranges with multiple fauna, and interactions between chimpanzees and sympatric species have been widely reported across African field sites (e.g., Hosaka and Ihobe 2015; Hockings et al. 2012). Across many sites “play bouts” have been reported between chimpanzees and sympatric species. However, in most cases, these interspecific play bouts appear non-mutual, and often involve the chimpanzee ‘player’ using interspecific ‘playmates’ as objects. Many of these reported cases result in the death of the ‘playmate’. In the wild, young chimpanzees in Taï Forest, Ivory Coast, have been observed engaging in non-mutual object play with both duikers (*Cephalophus *sp.; Boesch and Boesch 1989) and flying squirrels, (*Anomalurus derbianus;* Boesch and Boesch-Achermann 2000). Duikers are also occasionally preyed upon by these apes although squirrels appear to be neglected by Taï chimpanzees (Boesch and Boesch 1989). In at least one of these cases, the non-mutual play bout was reported to have ended with the death of the recipient (Boesch and Boesch 1989). Chimpanzees in Bossou, Guinea, have also been observed catching and playing with western tree hyraxes (*Dendrohyrax dorsalis*; Hirata et al. 2001) and African wood-owls (*Ciccaba woodfordi*; Carvalho et al. 2010), with no attempt at ingestion. In one of the cases, Hirata et al. (2001) observed an adolescent female chimpanzee carrying a dead hyrax that was killed by other members of the group, for 15 h, sleeping with it and grooming the corpse. Of the two chimpanzee–hyrax interactions (Hirata et al. 2001), one hyrax survived. Neither owl used for play survived. Of the other great apes, this kind of interspecies non-mutual play has also been reported in bonobos (*Pan paniscus*). A recently published anecdote reported a non-lethal interaction between a bonobo and a duiker at Wamba (Yokoyama 2021)*.* In this event, an adult female bonobo was seen carrying a living duiker around for 30 min without injuring it. The authors describe the behavior of the bonobo toward the duiker as characteristic of play but note that elsewhere duikers are a bonobo prey species.

Of the reported affiliative interspecific interactions *between* primates, affiliative interactions, particularly cross-species grooming events, remain relatively rare. The drivers and functions of this behavior are undetermined. Interspecies grooming events have been observed between a wide number of primate species in captivity and in the wild (summarized in Table 1). In the wild, outside of great apes, interspecies grooming events involving at least one primate participant have been observed between several species and can include non-primate recipients. Amongst wild great apes, interspecific grooming events with other primate species have only been reported in chimpanzees (Tsutaya et al. 2018; Bakuneeta 1996; John and Reynolds 1997) and bonobos (Sabater Pi et al. 1993; Ihobe 1990). A few cases of affiliative interspecific interactions involving chimpanzees and non-primate species have been described, both in the wild (Hockings et al. 2012) and in captivity (Ross et al. 2009), however, a cross-site compilation of reported interspecific grooming events in chimpanzees has not yet been published. Anecdotal reports of interspecies grooming between great apes and other primate species are notably underrepresented in the primatological literature (though see Tsutaya et al. 2018; Bakuneeta 1996; John and Reynolds 1997), however, there are likely many other observed cases of interspecies grooming including primate species that have remained unpublished, leading to an underreporting of this behavior.

**Table 1:** Interspecific grooming events involving at least one non-ape primate (in wild and captive contexts)

Context	Species 1	Species 2	Citations
Captive (Primate-Primate)	Moustached tamarins (*Saguinus mystax*)	Brown-mantled tamarins (*Saguinus fuscicollis*)	Heymann and Sicchar Valdez (1988)
	Long-tailed macaques (*Macaca fascicularis*)	Patas monkeys (*Erythrocebus patas*)	Baker and Preston (1973)
	Capuchin monkeys (*Cebus albifrons* and* Sapajus apella*)	Spider monkeys (*Ateles geoffroyi* and * Ateles paniscus*)	Maple and Westlund (1975)
Wild (Primate-Primate)	Raffles’ banded langurs (*Presbytis femoralis*)	Long-tailed macaques (*Macaca fascicularis*)	Lee et al. (2021)
	Grey langurs (*Semnopithecus priam thersites*)	Toque macaques (*Macaca sinica sinica*)	M.A. Huffman, unpublished observations
	Rhesus macaques (*Macaca mulatta)*	Hanuman langur (*Semnopithecus entellus*)	Nerlekar (2012)
	Red-tailed monkeys (*Cercopithecus ascanius*)	Blue monkeys (*Cercopithecus mitis*)	Gathua (2000)
	Southern bamboo lemurs (*Hapalemur meridionalis*)	Ring-tailed lemurs (*Lemur catta*)	Eppley et al. (2015)
	Red colobus (*Colobus badius*)	Red-tailed monkeys (*Cercopithecus ascanius*)	Struhsaker (1981)
	Red colobus (*Procolobus badius*)	Black-and-white colobus monkeys (*Colobus polykomos*)	Fimbel (1992)
	Abyssinian black-and-white colobus (*Colobus guereza*)	Red-tailed monkeys (*Cercopithecus ascanius*)	Struhsaker (1981)
Wild (Primate-Non-primate)	Japanese macaques (*Macaca fuscata*)	Spotted deer (*Cervus nippon*)	M.A. Huffman, personal communication
	Rhesus macaques (*Macaca mulatta*)	Sambar deer (*Rusa unicolor*)	Vasava et al. (2013)

To date, published anecdotes of chimpanzee interspecies grooming events are restricted to three reports: from Kalinzu Forest Reserve, Western Uganda (Tsutaya et al. 2018), Kaniyo-Pabidi community (Bakuneeta 1996) in Budongo Forest, Uganda and from the Sonso community (John and Reynolds 1997) also in Budongo Forest, Uganda. These events are summarized in Table 2. At Kalinzu, four cases of interspecies grooming have been reported, two of which involved female chimpanzees grooming adult, male, blue monkeys, and the other two involved female chimpanzees grooming adult, male, red-tailed monkeys (Tsutaya et al. 2018). In all cases at this site, an adult, male, monkey recipient approached and solicited grooming from a female chimpanzee groomer. In no case did the monkey recipient reciprocate. In three cases, monkey recipients solicited grooming from mother-infant chimpanzee pairs, and in the fourth case, the monkey approached a juvenile female who had been traveling with a nulliparous adult female. At Kaniyo-Pabidi, Bakuneeta (1996) observed an unidentified monkey following a group of chimpanzees. The monkey was observed feeding with and grooming the chimpanzees in this group. The monkey was also groomed by members of the group. The case recorded from the Sonso community in Budongo (John and Reynolds 1997) discussed later as observation 1 involves an adult, monkey recipient and a juvenile female chimpanzee groomer.

**Table 2:** Interspecific interactions involving at least one chimpanzee

Chimpanzee groomer (age, sex, species)	Recipient species (age, sex, species)	Field site	Citations
Adult, female, chimpanzee (mother–infant pair)	Adult, male, BM	Kalinzu	Tsutaya et al. (2018)
Adult, female, chimpanzee (mother–infant pair)	Adult, male, BM	Kalinzu	Tsutaya et al. (2018)
Adult, female, chimpanzee (mother–infant pair)	Adult, male, RTM	Kalinzu	Tsutaya et al. (2018)
Juvenile, female, chimpanzee	Adult, male, RTM	Kalinzu	Tsutaya et al. (2018)
Unspecified	Unidentified	Kaniyo-Pabidi (Budongo)	Bakuneeta (1996)
Juvenile, female, chimpanzee	Adult, unknown, RTM	Sonso (Budongo)	John and Reynolds ( 1997)

*RTM* red-tailed monkey, *BM* blue monkey

Amongst the other great apes, only bonobos (*Pan paniscus)* have been observed engaging in affiliative relationships with other primate species in the wild. In Wamba, DRC, Ihobe (1997) reported that guenons, including red-tailed monkeys (*C. ascanius*) and Wolf’s mona monkey *(C. wolfi*), were seen approaching bonobos without initiating direct contact, traveling, feeding, and resting together. In one case, also reported from the site, a young colobus monkey (*Colobus badius*) followed a group of bonobos for 18 consecutive days (Ihobe 1997). Bonobos are also the only other great apes who have been observed interspecies grooming with sympatric primates (i.e., Ihobe 1990, 1997; Sabater Pi et al. 1993; Yokoyama 2021). Bonobos from the Lilungu region of the Democratic Republic of Congo (DRC) engaged in affiliative and social activity with captured young colobus monkeys (*Colobus angolensis*) and red-tailed monkeys (*Cercopithecus ascanius*; Sabater Pi et al. 1993). In both reported cases involving interspecific interactions between bonobos and red-tailed monkeys (Sabater Pi et al. 1993), the bonobos groomed the red-tailed monkeys before subsequently killing them. In Wamba, DRC, in at least two cases, adult male colobus monkeys (*Colobus badius)* were also observed grooming bonobos (Ihobe 1990). Like chimpanzees, bonobos also hunt mammal species for meat, including sympatric primates, although hunting of other primates is relatively rare (but see Surbeck and Hohmann 2008). As far as the authors know, there are no published cases of interspecific grooming between wild great ape species (but see Sanz et al. 2022 for recent evidence of chimpanzee–gorilla play interactions).

In primates, allogrooming is defined as “caregiving through physical contact, typically where one animal uses its hands, mouth, or other part of its body to touch another animal” and usually occurs between members of the same species (Russell 2018: pp. 1). Allogrooming involves a minimum of two members of the same species (a groomer and recipient) (Lee et al. 2021) and can be both unidirectional and/or mutual. In chimpanzees, polyadic grooming is also common, occurring among triads or larger chains (Goodall 1986; Nakamura 2000; Girard-Buttoz et al. 2020). Allogrooming in primates has been shown to be multifunctional (Spruijt et al. 1992), allowing for the establishment and maintenance of social bonds (Lehmann et al. 2007) between kin (Schino and Aureli 2010) and non-kin conspecifics (Dunbar 1991; Goosen 1981; Crockford et al. 2013). Allogrooming also appears to improve hygiene by reducing external parasite loads in recipients (Akinyi et al. 2013, Mooring et al. 2004; Zamma 2002; Schino et al. 1988; Keverne et al. 1989; Tanaka and Takefushi 1993; Aureli et al. 1999; Radford 2012). Recipients of grooming can also benefit from stress reduction (Boccia et al. 1989; Shutt et al. 2007; Maestripieri et al. 1992; Schino et al. 1996) and thermoregulation (McFarland et al. 2016). However, allogrooming also has costs, including depletion of energetic budgets and opportunity costs (Dunbar 1992), potential proximity to aggressive conspecifics (Schino and Alessandrini 2015), and exposure to ectoparasites and infective stage endoparasites (Hernandez and Sukhdeo 1995; Veà et al. 1999; MacIntosh et al. 2012; Russell and Phelps 2013; Lee et al. 2021). While it is likely that interspecific grooming bouts also have benefits and costs, there are also likely fewer mutualistic advantages. However, site-specific anecdotes suggest possible explanations for this unusual behavior. While interspecies grooming could be a form of interspecies play for the chimpanzee groomers, for clarity, this paper will draw a distinction between interspecific ‘grooming events’ in which grooming appears to be the primary goal of the affiliative interaction and interspecific ‘play events’ which include varied non-aggressive behaviors such as chasing, slapping, and non-predatory physical contact.

We discuss seven events, six observations of interspecies social grooming, and one of interspecies play in the Budongo Forest recorded between 1996 and 2021. These occurred between East African chimpanzees (*Pan troglodytes schweinfurthii*) from the Sonso community and two individuals of the *Cercopithecus* genus. Five out of six of the reported interspecies grooming events and the interspecies play event occurred between chimpanzees and red-tailed monkeys (*C. ascanius*), while one interspecies grooming event occurred between a chimpanzee and a blue monkey (*C. mitis*). Both species of *Cercopithecus* are also known prey species for this chimpanzee community (Hobaiter et al. 2017; Newton-Fisher et al. 2002).

## Methods

### Study site and subjects

The Budongo Forest Reserve is a semi-deciduous tropical rain forest consisting of 793 km^2^ of protected forest and grassland, located along the western Rift Valley in Uganda. The Budongo Forest is a medium-altitude rainforest (~ 1100 m) with high annual rainfall (~ 1500 mm per year). A dry-season occurs between December–March followed by another, even drier season during June–August (Newton-Fisher 1999). The forest contains a population of approximately 600 East African chimpanzees. There are two habituated chimpanzee communities: the Sonso community (since 1990) and the Waibira community (since 2011). In addition to chimpanzees, four other species of primate are regularly observed within the Sonso and Waibira home ranges, including Olive Baboons (*Papio anubis*), Blue Monkeys (*Cercopithecus mitis*), Red-tailed monkeys (*Cercopithecus ascanius*), and Black and White Colobus monkeys (*Colobus guereza*). The six observations recorded in this study took place in the Sonso community. At the end of the observation period in 2021, the community was considered a typical size (~ 69 individuals; Wilson et al. 2014) and had a typical female-biased sex ratio among mature individuals (M:F; 1:1.7).

### Ethical note

All data collection in this study were observational and adhered to the International Primatological Society’s Code of Best Practice for Field Primatology (Riley et al. 2014). Researchers adhered to all applicable international, national, and institutional guidelines for the care of animals. Research was approved by the Uganda Wildlife Authority (UWA) and the Uganda National Council for Science and Technology (UNCST). All work met the ethical standards of the Budongo Conservation Field Station where the observations were made. The authors declare that they have no conflicts of interest.

### Data availability

Video of one of these events (observation 6: 9/2021) is available in the supplementary materials.

### Data collection

Researchers and field assistants (hereby referred to as field colleagues) follow chimpanzees in Sonso daily from 07:00 to 16:30. Long-term data collection, recorded by field colleagues, includes focal individual activity and party composition taken on a 15-min scan basis. In addition, when unusual events occur, event details are recorded into the station logbook. Types of events added to these books include (but are not limited to) births, deaths, intercommunity killings, respiratory disease outbreaks, unusual feeding behaviors, and hunts. As far as the authors know, there were no major gaps in data collection. However, it is possible that not all interspecies grooming events were recorded, as this behavior may not have always been considered a behavior of interest by past researchers or field colleagues.

### Results

Most of the observations analyzed below come from in the Sonso logbook, which contains events dating back to 1993. Observation 1 was previously published by John and Reynolds (1997). Observation 5 was not written down due to data collection disruption during the COVID-19 pandemic. This event was later transcribed post hoc from GM’s field notebook. Table 3 summarizes all observations of interspecific grooming and interspecific play events recorded thus far amongst members of the Sonso community in Budongo Forest. Unfortunately, neither interspecific interaction nor opportunities for interspecific interaction are systematically recorded in the long-term data, making it impossible to calculate what proportion of interspecies group events result in affiliative (or agonistic) interspecies interactions.

**Table 3:** Summary of interspecific grooming and interspecific play events between Sonso chimpanzees and other primate species

Obs. no.	Chimpanzee actor (s)	Behavior	Month/Year	Age class and sex of actor	Recipient species	Age class and sex of recipient	Event duration	Ecological context
1	Gonza (GZ)	Groom	09/1996	Juvenile (6 years old ± 1 year), female	RTM	Adult Unknown	~ 25 m	Feeding in *Khaya anotheca* tree
2	Kumi (KM)	Groom	10/2002	Infant (4 years old), female	BM	Unknown Male	Unknown	Feeding in *Broussonetia papyrifera* tree
3	Karo (KR)	Groom	01/2006	Juvenile (5 years old), female	RTM	Unknown Unknown	~ 1 h 5 m	Feeding in *Ficus sur* tree
4	Karo (KR)	Groom	12/2007	Juvenile (6 years old), female	RTM	Unknown Unknown	Unknown	Feeding in *Broussonetia papyrifera* tree after eagle sighting
5	Ishe (IS)	Groom	04/2021	Infant (4 years old), female	RTM	Adult Male	Unknown	Feeding in mango tree
6	Ishe (IS) and Dembe (DB)	Groom	09/2021	Infant (4 years old), female Infant (3 years old), female	RTM	Adult Male	~ 14 m	Feeding in *Croton sylvaticus* tree
7	Muhumuza (MZ), Kaija (KJ), and Kefa (KF)	Play	10/2017	Infant (2 years old), male Infant (4 years old), Male Infant (3 years old), male	RTM	Male	20 m	Feeding in *Broussonetia papyrifera* tree

*RTM* red-tailed monkey, *BM* blue monkey

#### Observation 1: September 1996, Gonza grooms a red-tailed monkey

On September 4, 1996, a group of chimpanzees, Musa (adult male), Kewaya (adult female), Zimba (adult female), and her offspring Gonza (sub-adult female) were observed together, feeding in different trees approximately 7–15 m apart. At 08:42, Gonza was seen alone in the southwest of a *Khaya anotheca* (KA) tree watching an adult red-tailed monkey who was also resting in the same tree. Gonza approached the monkey until she was ~ 3 m away, and then shook a branch in the monkey’s direction. The monkey, however, remained resting and did not move or appear agitated by this display. Gonza repeated the branch shaking behavior three times and then moved closer to the monkey, who was facing away from her. Gonza grabbed the monkey’s tail and started to shake it, in a manner that appeared to be playful, folding the tail around her neck and then shaking it again. This lasted for ~ 2 min. At 08:47, Gonza attempted to groom the monkey below the anus, which the monkey seemed to welcome, positioning its legs to give Gonza access. She groomed the monkey under the abdomen, chest, and back, interspersing grooming with play-like behaviors, including hitting the monkey’s sides, and pulling the legs. At one point, Gonza appeared to rub her genitals against the anus of the monkey. The grooming event lasted 20 min and at no point did the monkey reciprocate grooming. At 09:07, the monkey moved away, ending the grooming session. Gonza tried to follow, but the monkey quickly moved to another tree out of reach. Gonza returned to her mother Zimba.

*Observation reported by Kakura John, Jachan G., and Tinka John*

#### Observation 2: October 2002, Kumi grooms a blue monkey

On October 1, 2002, Kalema’s female infant Kumi was seen grooming a solitary male blue monkey in the presence of four other chimpanzees. At 15:23 Kumi was seen moving close to the lone male monkey, who looked relaxed while feeding on the flowers of *Broussonetia papyrifera*. Kumi touched the tail of the monkey, but the monkey did not move or appear disturbed. At 15:24, she played with the tail of the blue monkey. After this, she touched the monkey’s testes, and then smelled her hand. At 15:25, Kumi resumed grooming the monkey. At 15:26, Kumi stopped grooming and moved away, ending the interaction.

*Observation reported by Geresomu Muhumuza*

#### Observation 3: January 2006, Karo grooms a red-tailed monkey

On the morning of January 3, 2006, Karo, a juvenile female, was observed grooming a red-tailed monkey. A group of at least 14 chimpanzees were feeding on the fruits of *Ficus sur,* including seven adult males, six adult females, and one sub-adult female. A red-tailed monkey joined them. The chimpanzees were high up above the monkey who began feeding below them. At 08:07, Karo approached the red-tailed monkey who laid down and presented his face to Karo, who then groomed him. At 09:09, Kalema, (adult female) approached Karo and the monkey. In response, the monkey moved approximately 6 m away, where he stopped and continued to feed. At 09:10, Karo once again approached the monkey and resumed grooming him. At 09:12, Musa (adult male) approached the monkey, and the monkey jumped out of the *Ficus sur* tree and into another tree nearby.

*Observation reported by Monday Mbotella Gideon and Jackson Okuti*

#### Observation 4: December 2007, Karo grooms a red-tailed monkey

**Fig. 1 and 2:**
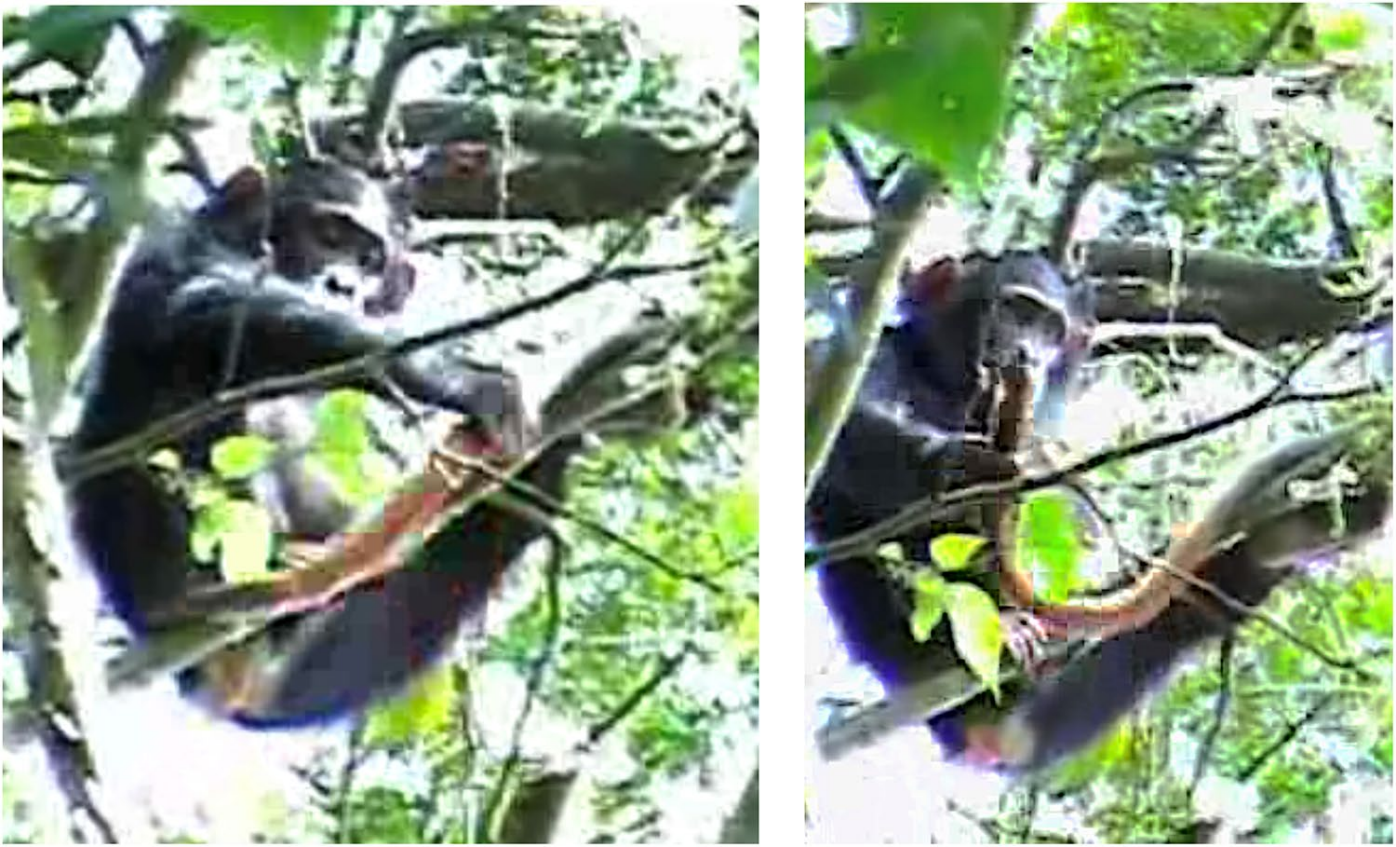
Karo plays with the tail of a red-tailed monkey after grooming (Photo by CH)

On December 2, 2007, at 09:32, Karo approached a lone red-tailed monkey who was resting in a *Broussonetia papyrifera* tree. This occurred directly following a disturbance caused by a crown eagle flying overhead, which had occurred at 09:13. Nearby colobus monkeys, blue monkeys, and red-tailed monkeys, upon seeing the eagle, had all begun vocalizing and dispersed in different directions. The lone red-tailed monkey, however, had remained in the tree. Karo approached the monkey and started playing with his tail. She then groomed him. Karo also inspected his backside and testes with her finger, smelled it, and then then wiped the finger on a branch. When Kalema (adult female) who was nearby started to leave, Karo ended the grooming bout and followed her. Kalema and Karo moved southwest to join the rest of the group ~ 200 m away. The red-tailed monkey moved off alone. There were no other red-tailed monkeys around (see Figs. 1 and 2).

*Observation reported by Catherine Hobaiter and Amati Stephen*

#### Observation 5: April 2021, Ishe grooms a red-tailed monkey

In late April (exact date and time unknown) Ishe, an infant female chimpanzee, was observed grooming an adult, male red-tailed monkey. Ishe and the monkey were both sitting in a mango tree by the abandoned schoolhouse. The monkey began moving closer to Ishe, presenting his head. When she began grooming him, he turned to the side, and she groomed him there as well. Then he turned and presented his back. Ishe appeared interested in his tail and rolled it around her own neck. Irene (adult female), Ishe’s mother, moved closer to the pair, and the monkey ran away. The grooming bout’s duration was not recorded.

*Observation reported by Geresomu Muhumuza*

#### Observation 6: September 2021, Ishe grooms a red-tailed monkey and Dembe plays with his tail

**Fig. 3:**
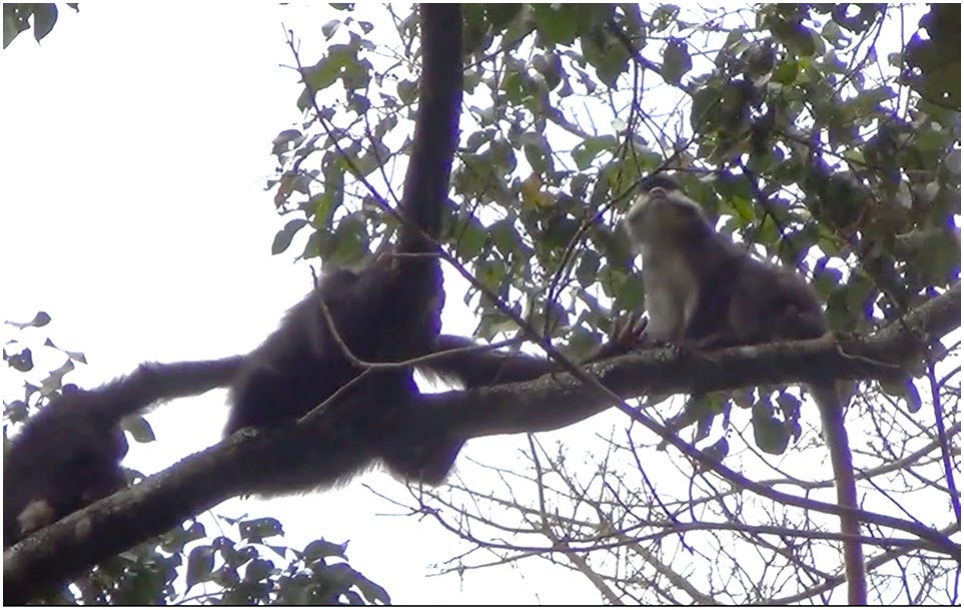
Ishe reaching toward a red-tail monkey while Dembe grooms Ishe (Photo by EF)

**Fig. 4:**
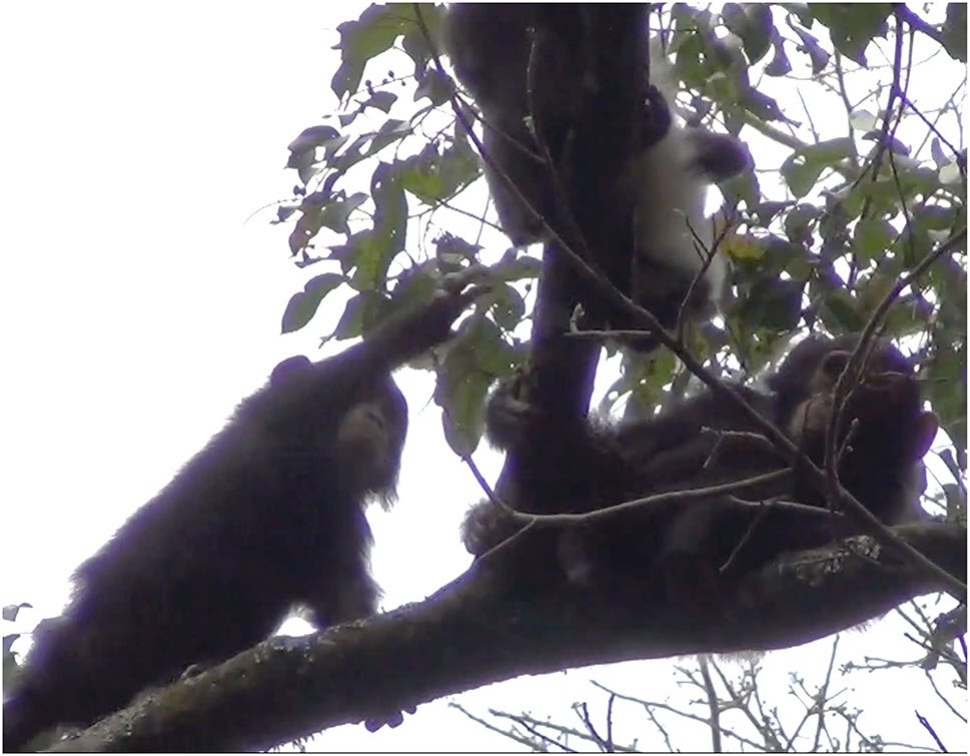
Ishe reaching toward a red-tail monkey while Dembe watches (Photo by EF)

On September 3, 2021, Ishe groomed a red-tailed monkey (See Supplementary Materials). At 10:05, Ishe (infant female) and Dembe (infant female) were observed in a *Croton sylvaticus* tree (CSY), eating fruits, while their mothers (Irene and Deli) remained in the nearby *Ficus variifolia* (FVR). At 10:18, an adult male red-tailed monkey crossed into the CSY. Ishe approached him cautiously and then turned to present her back to him. She then turned and appeared to groom the monkey. The monkey sat upright and then turned his back to her. She groomed the back of his hind legs. While Ishe was doing this, Dembe approached but stayed behind Ishe, and then retreated. The monkey moved higher in the tree and lay down. Dembe approached again and touched the monkey’s neck, then smelled her hand. This happened twice. Dembe retreated and the monkey stood quadrupedally, presenting his backside to Ishe. Dembe began to groom Ishe. The monkey turned to face Ishe again and Ishe put her hand out, moving her fingers in a beckoning motion to the monkey. Ishe turned her back to the monkey and he crossed over to her but did not groom her. He then moved off but remained close by. The red-tailed monkey did not appear scared of Ishe or Dembe. Dembe seemed hesitant about approaching the monkey but appeared to gain confidence after watching Ishe. After the red-tailed monkey moved, the two infants continued feeding. While they fed, Ishe shook a branch at the monkey a couple more times. A few minutes later, Ishe approached him again, shaking a branch in his direction. The monkey continued feeding and moved a few meters below. Dembe came to join Ishe. Dembe moved closer to the monkey, whose tail was extended upward toward her. Dembe extended a hand and grabbed the monkey’s tail, slightly swinging the tail and pulling it for around 10 s, while the monkey continued feeding. At 10:32, the monkey moved away and Dembe went to join Ishe. At 10:35, Dembe moved out of the tree, and Ishe remained with the monkey. She stomped on the branch she was sitting on at 10:35, and the monkey did not react. Ishe then crossed and connected back into the FVR. No other red-tailed monkeys were seen or heard during the observation (see Figs. 3 and 4).

*Observation reported by Elodie Freymann and Geresomu Muhumuza*

#### Observation 7: October 2017, Kefa, Muhumuza, and Kaija engage in interspecific play with a red-tailed monkey

At 09:36, in blocks 2–1 and 2–0, while watching chimps feeding on flowers of *Broussonetia papyrifera (BPY)*, an adult male red-tailed monkey approached three infant males (Kefa, Muhumuza, and Kaija) as they were playing in a BPY tree. The monkey presented its back first to Kefa who instead of grooming, slapped the monkey, and then Muhumuza grabbed the monkey’s tail, and Kaija reached his hands to the mouth of the monkey. At one point, they started chasing each other through the canopy and the monkey followed them. The play bout lasted for about 20 min.

*Observation reported by Monday Mbotella Gideon, Mourean (veterinary intern*)

## Discussion

In total, six cases of interspecies grooming, and a single case of affiliative interspecies play (involving no unidirectional or mutual grooming) with another primate species have been recorded in the Sonso chimpanzee community. These six cases add to the growing record of chimpanzee-sympatric primate interspecies grooming events reported at wild chimpanzee field sites. As far as the authors know, there have been no published cases reported outside of Uganda. Of the six interspecies grooming events from Budongo, five involved red-tailed monkeys and one involved a blue monkey. In at least five of the grooming events, playing with the monkey’s tail was recorded. Examination of monkey tails appears to be relatively common amongst chimpanzees, especially amongst younger individuals, and infants have been observed playing with the tails of prey after a hunt (A. Mielke, personal communication). In observations 1–4 and 6, the chimpanzee groomer appeared to initiate the interaction event by approaching the monkey, while in observation 5 the red-tailed monkey initially approached the chimpanzee. In the previously reported interspecific grooming events from Kalinzu, like in observation 5, the monkey recipient is reported to have approached the chimpanzee groomer. In all cases, it remains difficult to confirm which individual initiated the grooming bout itself.

In all six observations of interspecies grooming the chimpanzee was a female between the ages of 4–6 years old, while the single case of play (with no grooming) included three infant males. This apparent sex bias towards female interspecific groomers is consistent with the four reported cases from Kalinzu (in which all chimpanzee groomers were female). As the reported cases from Kaniyo-Pabidi do not specify the sex of the groomer, this cannot be assessed. While two of the groomers in Kalinzu were adults, both had young offspring present, and the other two cases involved juvenile groomers. While the sample size limits interpretations, this fits with the tendency described in Gombe for immature female chimpanzees to groom conspecifics more frequently, while immature males tend to play with conspecifics more than females (Lonsdorf et al. 2014; Meredith 2013; Lonsdorf 2017). Why the monkeys approached females rather than male juveniles to solicit grooming in the above cases remains unexplained. In two of the interspecific grooming cases (observations 2 and 4), the female chimpanzee appeared to touch the testes of the receiving male monkey, and then smelled her fingers. In a third case (observation 6) the female chimpanzee groomer touched the neck of the receiver and then smelled her fingers. This suggests that there could be an additional olfactory or hormonal cue that the chimpanzee groomer is interested in or sensitive to.

If the guenons were the initiators of these events, their potential preference for juvenile grooming partners may be explained by chimpanzee hunting patterns. In Budongo, both red-tailed monkeys and blue monkeys are hunted by Sonso chimpanzees, although blue monkeys appear to be the more popular prey target. Between 1999 and 2017, Hobaiter et al. (2017) reported 23 hunting attempts on blue monkeys, and only seven attempts on red-tailed monkeys. As most hunts of guenon species are carried out by adult chimpanzees (Ross et al. 2009), adult red-tailed monkeys may feel unthreatened approaching or being approached by smaller-bodied juveniles. Even though young chimpanzees do not hunt monkeys, they may still be strong enough to injure or even kill them through rough play. However, the monkey recipients in the cases described appeared to act as if there was little risk of a fatal attack or dangerous play behaviors. Similarly, the chimpanzee groomers must have had some level of understanding to adapt their grooming and play style to the physical strength of the recipient species. As guenons have sharp canines, the monkey recipients could pose a threat to infant and juvenile chimpanzees, despite their smaller size.

If, in the above cases, the juvenile chimpanzees were the primary initiators of interspecific grooming events, this would be consistent with findings that in both wild and captive chimpanzees, non-fatal and non-consumptive interspecific interactions are mostly carried out by juveniles or early adolescents (Hockings et al. 2012; Teleki 1973; Goodall 1986; Boesch and Boesch-Achermann 2000; Ross et al. 2009). Immature chimpanzees in Bossou were significantly more likely than adults to engage in play with other species, and adults never engaged in playful interactions with other species (Hockings et al. 2012). Interspecific play and grooming by juveniles, therefore, could occur as practice for conspecific grooming and exploration—using animals they see frequently, and with which they share some similar biological characteristics, to hone their skills during this critical learning period. However, a possibly more parsimonious explanation is that at this age, chimpanzee juveniles do not discriminate other species into prey and playmate categories. Their playful nature may allow them to engage in affiliative interactions with other nearby individuals, regardless of species.

Across chimpanzee field sites, red-tailed monkeys appear to be the most common receivers of affiliative interspecific events, although this apparent species bias may be an artefact if red-tailed monkeys have become more habituated to human researchers at these field sites than other primate species, and are thus more easily detected in these interactions. A detection bias may also be due to the density of red-tailed moneys at chimpanzee field sites and their potential overlap with chimpanzees regarding feeding ecology or active hours. In the cases reported from Kalinzu, red-tailed monkeys seem to be repeated receivers of interspecific grooming from chimpanzees. In Gombe, chimpanzees were also reported to have played with a red-tailed monkey, and in Mahale the chimpanzees of the M-Group showed tolerance toward red-tailed monkeys as they co-fed. Blue monkeys were also recipients of chimpanzee grooming at two sites at least (two cases from Kalinzu and one case from Budongo [observation 2]). While we do not have data on its frequency, observations of co-feeding events, in which groups of chimpanzees peacefully co-feed with either red-tailed or blue monkey individuals in the same tree are not uncommon, occurring perhaps several times a month depending on the food species available. While these anecdotal observations cannot be used to calculate a proportion of how many opportunities for interspecific interactions result in interspecies grooming, regular neutral interspecific interactions, such as co-feeding between chimpanzees and both *Cercopithecu*s species, appear to be present in Budongo.

Interestingly, two juvenile females (Karo and Ishe) were both observed engaging in interspecific grooming at least twice each, suggesting that these individuals may have had a preference or proclivity for interspecies grooming behaviors or, if the events were initiated by the monkeys, that they were targeted as favorable grooming partners. While this could reflect individual preferences or personality traits in the chimpanzee groomers or the monkey receivers, it could also be a result of socially learned or socially facilitated interspecies affiliative partner selection. If both chimpanzee subjects were exposed to interspecies grooming at a young age (through observations of experienced individuals engaging in this behavior), they may be more likely to seek out opportunities to groom other species themselves, which might account for the appearance of an individual-level preference for the behavior (Hockings et al. 2012). While this may explain the repeated observations of certain individuals engaging in interspecies grooming, this hypothesis cannot be tested without a larger sample size.

Of these interspecific grooming bouts, cases were largely observed during the wetter seasons, with only one case occurring at the very beginning of a dry season (observation 4). These results suggest that fruit scarcity, and thus competition, is likely not a driver of forced interspecific interactions; as was the case with orangutan and red leaf monkey polyspecific associations (Hanya and Bernard 2021), as fruits are widely available during Budongo’s wet season, and there are plenty of trees simultaneously bearing fruit. However, more limited competition could also facilitate interspecific grooming events, as the subsequent reduction in stress due to abundant fruit may eliminate the need for chimpanzees to act agonistically toward other potential competitors. Seasonal variation in hunting frequency has also been suggested amongst Budongo chimpanzees (Hobaiter et al. 2017); however, not enough information is available to determine whether periods with low hunting rates may correspond to periods of increased interspecies grooming. Furthermore, the diversity of tree species in which these cases were observed (*Ficus sur, Croton sylvaticus, Broussonetia papyrifera* (2),* Magnifera* sp.,* Khaya anotheca*) suggests that a specific ecological context is also not necessarily a driver of interspecies grooming. However, as the diets of guenon species are still understudied in Budongo, dietary overlap cannot be ruled out as a factor affecting chimpanzee–guenon interaction rates or competition (but see Wrangham et al. 1998 for comparative study on primate diets in Kibale National Park).

*Do chimpanzees benefit from unidirectional interspecific grooming bouts at Budongo or is it a form of object play? And why do solitary guenon males appear to spontaneously approach isolated mother–offspring pairs or solitary infant/juvenile female chimpanzees?*

One-way interspecific grooming by chimpanzees likely has multiple costs. For one, there are energetic costs to grooming itself and grooming slows down feeding efficiency (Russell and Phelps 2013). Being in close contact with another species may also increase chimpanzees’ exposure to parasites or zoonotic illness, including novel pathogens, which may pose hygienic threats, and other harmful microbiota (Moeller et al. 2013). There is also likely a social opportunity cost to interspecies grooming, as the groomers’ time could otherwise be spent grooming conspecifics and strengthening affiliation with members of their own group. Instead, the chimpanzee groomers “spend” that social investment on a species that does not *directly* appear to return the favor. However, there could possibly be long-term, indirect advantages, such as possibly benefiting from the arboreal monkey’s vigilance to avoid risk more effectively (i.e., from snakes, hunters, and other anthropogenic disturbances). In at least two of the six observations of interspecies grooming, multiple juveniles were present when the interspecific grooming event took place, and in all cases the juvenile’s mother was nearby. This is also true of the interspecific play bout (observation 7). For each of these cases, there may have therefore been some social opportunity cost to the interspecies grooming bout. However, if in these cases the chimpanzee groomers regarded the interspecies receivers as merely play objects, it could be that play with a monkey is better than no play at all or offers an alternative ‘novel’ source of engagement. Furthermore, the interspecies grooming bout involving two infants and a red-tailed monkey (observation 6) could also promote conspecific social bonding between the chimpanzee infants.

Past papers on polyspecific associations in primates have suggested that interspecies grooming bouts could promote coalition or alliance across species, suggesting that interspecific group merging may increase group size and deter predation (de Carvalho Oliveira et al. 2017; Hanya and Bernard 2021). There would be an incentive for red-tailed or blue monkeys to stay near chimpanzees in feeding trees if other predators such as eagles were nearby and posed a greater threat than the chimpanzees. However, this hypothesis seems an unlikely explanation for interspecies grooming on the side of the Sonso chimpanzees, who would likely not immediately benefit from predator deterrence. Interspecies grooming events also appear to occur too infrequently to be a long-lasting coalitionary behavior.

It is easier to identify possible benefits for the red-tailed monkeys, so they may simply be tolerated by adults and pose a source of amusement for the young chimpanzees. One possible benefit to the monkeys is hygiene. Blue monkeys and red-tailed monkeys are both highly susceptible to ticks and other ectoparasites (Freeland 1981). Unlike chimpanzees who spend much of their day allogrooming, blue monkey males migrate from their natal groups at puberty and outside the breeding season, and there is usually only one resident male per group (Cords 2000). Similarly, adult, male, red-tailed monkeys are intolerant of each other and do not form “bachelor groups” (Struhsaker 1980; Butynski 1982). Tsutaya et al. (2018) proposed that solitary male, red-tailed and blue monkeys may approach mother–offspring chimpanzee pairs to receive grooming necessary to maintain their hygiene. If true, interspecies grooming could potentially be viewed as a form of currently undescribed interspecies health maintenance behavior (sic. Huffman 1997). Struhsaker (1981) reported that lone, male, red-tailed monkeys have been observed traveling with groups of red colobus (*Colobus badius*) in the Kibale Forest, Uganda, and have been recipients of interspecific grooming. Detwiler (2002) also reported that in Gombe National Park, blue monkey and red-tailed monkeys hybridized and formed mixed groups, traveling, mating, and grooming with one another. Struhsaker (1981) also reported an observation of a solitary male red-tailed monkey traveling with and being groomed by Abyssinian black-and-white colobus in the Kalinzu Forest Reserve. However, this hypothesis is complicated by the fact that in five of the six cases reported here, the chimpanzee groomers appeared to have been the initiators of the grooming bouts, approaching the tolerant guenons, and that interspecies grooming seems too infrequent to make a substantial impact on guenon health status. To test this hypothesis, further research should be done on the seasonality of ectoparasite loads in non-human primates (i.e., Klein et al. 2018) and grooming patterns amongst peripheral, male guenons to see whether there are periods more likely than others when such interactions could be more beneficial.

The small number of reported interspecies grooming events at Budongo, as well as the dearth of reported cases in the primatological literature, suggests that interspecies grooming is likely a rare behavior amongst wild chimpanzees. However, it is likely that reporting bias could contribute to this underrepresentation. Many observations of interspecies grooming or play events are not recorded or filmed due to lack of targeted research on these behaviors. The six cases of chimpanzee interspecies grooming reported here may be only a few of many cases that have occurred at Budongo and across other chimpanzee field sites. It is essential that anecdotal evidence from primate field sites be shared not only to encourage cross-site comparisons, but also to avoid losing valuable information about the behaviors of our closest primate cousins. Reporting these affiliative interactions between primate species can also reveal which species depend on each other in any given habitat and help prevent or predict ripple effects of extinction or endangerment. To better understand how rare this behavior is across chimpanzee field sites, future studies could survey site directors to determine whether attention is paid to interspecific affiliative interactions, and if so, how these events are recorded.

Collecting quantitative data on affiliative interactions will also be crucial to further understanding cross-species relationships between sympatric primates. Future study into interspecies affiliative interactions may also contribute useful context to the field of paleoanthropology, adding new ways of interpreting species proximity in the fossil record. Chimpanzee field sites should consider codifying interspecies interactions into their long-term data collection methods to begin gathering data which will allow researchers to quantify these behaviors more accurately. Many questions remain unanswered about interspecies grooming. *Why are some interspecies interactions affiliative, while other interactions with the same species are neutral or agonistic (predator–prey relationship)? What is the adaptive function for the ‘groomer’ in interspecies one-directional grooming events? Are interspecies grooming behaviors and preferences for this behavior socially learned?* The importance of collecting and publishing anecdotes remains paramount, as does communication between field sites and field researchers.

## Supplementary Information

Below is the link to the electronic supplementary material.

Supplementary file1 (MP4 305190 KB)

## Data Availability

All data are available upon reasonable request.
